# Supplementary figures and images for: Genomics-Based Reconstruction and Predictive Profiling of Amino Acid Biosynthesis in the Human Gut Microbiome
Source: Microorganisms. 2022 Mar 30;10(4):740. doi: 10.3390/microorganisms10040740 (PMC9026213; doi:10.3390/microorganisms10040740)

### Phenotype variability (genus level)

3 Number of Variable Phenotypes (NVP)

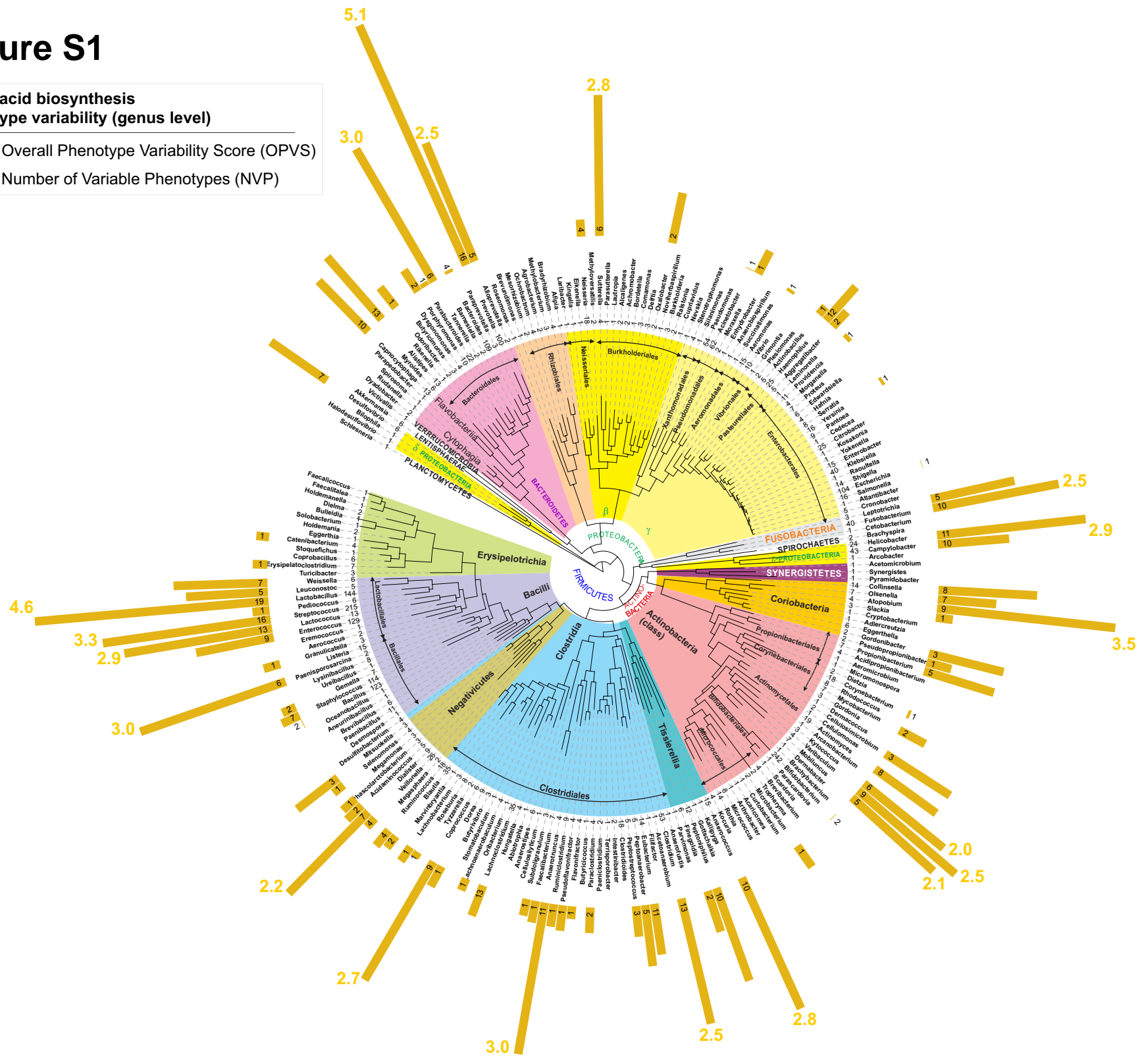

Supplement: Supplementary file 1 [file microorganisms-10-00740-s001.zip › Figure_S1.pdf]

Figure S2

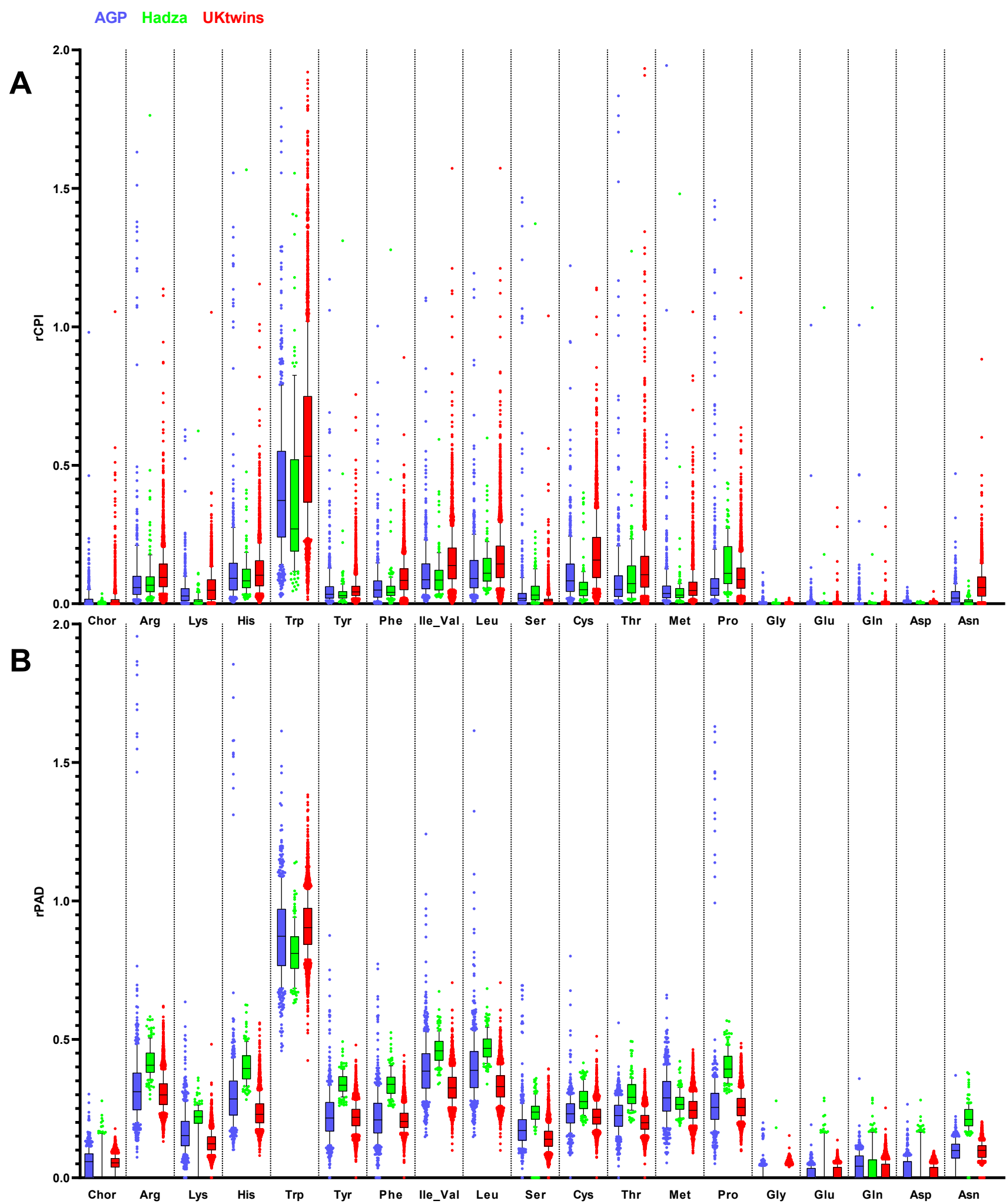

Supplement: Supplementary file 1 [file microorganisms-10-00740-s001.zip › Figure_S2.pdf]
